# Network Topologies and Convergent Aetiologies Arising from Deletions and Duplications Observed in Individuals with Autism

**DOI:** 10.1371/journal.pgen.1003523

**Published:** 2013-06-06

**Authors:** Hyun Ji Noh, Chris P. Ponting, Hannah C. Boulding, Stephen Meader, Catalina Betancur, Joseph D. Buxbaum, Dalila Pinto, Christian R. Marshall, Anath C. Lionel, Stephen W. Scherer, Caleb Webber

**Affiliations:** 1 MRC Functional Genomics Unit, University of Oxford, Department of Physiology, Anatomy, and Genetics, Oxford, United Kingdom; 2 INSERM U952, Paris, France; 3 CNRS UMR 7224, Paris, France; 4 UPMC Université Paris 06, Paris, France; 5 The Seaver Autism Center for Research and Treatment, Department of Psychiatry, Mount Sinai School of Medicine, New York, New York, United States of America; 6 Departments of Psychiatry, and Genetics and Genomic Sciences, Seaver Autism Center, The Mindich Child Health & Development Institute, Mount Sinai School of Medicine, New York, New York, United States of America; 7 The Centre for Applied Genomics and Program in Genetics and Genome Biology, The Hospital for Sick Children and the McLaughlin Centre and Department of Molecular Genetics, University of Toronto, Toronto, Ontario, Canada; Georgia Institute of Technology, United States of America

## Abstract

Autism Spectrum Disorders (ASD) are highly heritable and characterised by impairments in social interaction and communication, and restricted and repetitive behaviours. Considering four sets of *de novo* copy number variants (CNVs) identified in 181 individuals with autism and exploiting mouse functional genomics and known protein-protein interactions, we identified a large and significantly interconnected interaction network. This network contains 187 genes affected by CNVs drawn from 45% of the patients we considered and 22 genes previously implicated in ASD, of which 192 form a single interconnected cluster. On average, those patients with copy number changed genes from this network possess changes in 3 network genes, suggesting that epistasis mediated through the network is extensive. Correspondingly, genes that are highly connected within the network, and thus whose copy number change is predicted by the network to be more phenotypically consequential, are significantly enriched among patients that possess only a single *ASD-associated* network copy number changed gene (*p* = 0.002). Strikingly, deleted or disrupted genes from the network are significantly enriched in GO-annotated positive regulators (2.3-fold enrichment, corrected *p* = 2×10^−5^), whereas duplicated genes are significantly enriched in GO-annotated negative regulators (2.2-fold enrichment, corrected *p* = 0.005). The direction of copy change is highly informative in the context of the network, providing the means through which perturbations arising from distinct deletions or duplications can yield a common outcome. These findings reveal an extensive ASD-associated molecular network, whose topology indicates ASD-relevant mutational deleteriousness and that mechanistically details how convergent aetiologies can result extensively from CNVs affecting pathways causally implicated in ASD.

## Author Summary

Autism Spectrum Disorders (ASD) are characterised by impairments in social interaction and communication, and restricted and repetitive behaviours. ASD are highly heritable and many different stretches of DNA have been found to be duplicated or deleted in individuals with ASD. We found that an unusually high number of genes affected by these DNA deletions/duplications are associated with the functioning of synaptic transmission between nerve cells. The proteins made by many of these genes are known to interact with each other and, together with proteins from other deleted/duplicated genes, form a large interlinked biological network. This network was affected by almost 50% of the deletions/duplications in the ASD patients considered. Many individual ASD patients had deletions or duplications of multiple genes within this network, but for those patients with just a single gene from the network changed, that single gene appeared to play an important role. Furthermore, the network predicts that the effects arising from the genes in the deletions are similar to the effects arising from the genes in the duplications. Thus, the way that this ASD-associated network is wired together contributes to the understanding of the impact of these DNA deletions and duplications.

## Introduction

Autism Spectrum Disorders (ASD) form a group of complex disorders affecting ∼1% of individuals [1]. ASD are characterised by impairments in social interaction, communication, and restricted and repetitive interests and behaviours [2], although other symptoms such as intellectual disability, seizures or auditory abnormalities frequently co-occur [3]. Despite the high estimates of heritability for ASD found from monozygotic twin studies (∼90%) [4], the genetic cause is recognized in only ∼20% of cases suggesting that there are many causal variants yet to be identified [5], [6]. ASD-causative alleles are likely to be rare as (*i*) they are under strong purifying selection from the population due to the low fertility (∼5%) of individuals with ASD [7], and (*ii*) there is a strong positive correlation between paternal age and ASD risk which suggests that ASD-contributing mutations frequently may be arising *de novo* in the continuously-replicating paternal germ line [8]. Thus, in this study we examine *de novo* variants, specifically *de novo* copy number variants (CNVs), found in individuals with ASD as a set of variants likely enriched in causal mutations [6].

By contrast to methods that require either recurrent or common genetic variation to identify disease-associated loci, functional enrichment analysis (FEA) approaches gain considerable power by simultaneously examining the contributions of many disparate variants across many individuals' genomes and thus may be particularly appropriate for investigating the many rare and distributed variants underlying autism [9], [10]. FEA approaches hypothesise that dispersed variants observed in patients with shared symptoms may be affecting genes that participate in a common biological process and it is the disruption of the same process within each of these patients that underlies their common symptoms [11], [12]. Thus, FEA considers whether there is a functional category that is exceptionally common for genes overlapped by dispersed CNVs identified in the genomes of patients that present the same disorder. It thus associates function with the disorder and nominates those copy number variable genes that participate in that function as candidate disease genes [9].

The functional category types used in FEA approaches are key to the biological insights that they can provide. Different approaches have been applied to investigate the genetics underlying autism, including literature annotations [6], [13], protein-protein interactions [14], [15], mouse model phenotypes [13], gene co-expression [16] and functional linkage networks [6], [17]. As the application of these different approaches in autism studies often accompanies the publication of a novel genetic dataset, each method has highlighted many, usually novel, candidate genes that add to a rapidly growing list [18] and replication of significant functional enrichments has only rarely been attempted, let alone achieved [13], [19]. Synaptic functioning has been recurrently associated with ASD by many of the recent studies [13], [17] but the small proportions of genes that form these associations along with the functional diversity broadly exhibited by genes implicated in ASD has led authors to question specific associations [20]. However, given that it appears likely that the variants of several hundred genes contribute to autism [6], identifying those biological process(es) that are commonly disrupted may provide a more explanatory approach than to collate individual causative genes. In particular, FEA, when applied to ASD CNVs, should not just aim to identify unifying functional themes but should also provide a framework for interpreting how these variants exert their proposed phenotypic effects.

In this study, we examined the genes affected by four previously-published sets of rare, *de novo* CNVs identified in autistic patients. Given that ASD is a behavioural disorder, we initially considered the phenotype-level gene associations provided by mouse gene models before moving on to consider more molecular gene descriptions. We identified a significant enrichment of genes whose orthologues' disruption in mouse yields an *abnormal synaptic transmission* phenotype in 3 of 4 sets. We show that the protein products of the genes contributing to these enrichments form an extensive physical interaction network with genes previously implicated in autism and that extends to many other genes located in CNVs (herein termed CNV genes). We show that many of the autistic individuals considered here possess multiple CNV genes that reside within the network, suggesting extensive epistasis, and provide evidence that the number of interactions a gene has within the network is related to the propensity of its copy change to cause autism. Finally, within this network we find that whereas genes deleted in ASD are significantly enriched in those that positively regulate biological processes, the converse is also true: genes that are duplicated are enriched in negative regulators of biological processes. We provide several examples of how the direction of copy number change reinforces the biological interpretation of the ASD-associated physical interaction network.

## Results

Initially, we sought to objectively identify autism-related mouse behaviours among phenotypes that are over-represented among *de novo* CNV genes for individuals with ASD (herein termed ASD *dn* CNVs). For this, we obtained 4 sets of ASD *dn* CNVs (Table S1 and Materials and Methods). The set collated by the AGP is likely to be most powerful due both to its higher number of CNVs and their generally smaller sizes, although all methodologies employed in this study account for variations in the numbers of genes affected by each CNV set (Table S2). We considered *Gain* or *Loss* CNVs separately, in addition to the set formed from their union (*All*).

### Many mouse model phenotypes are associated with ASD *dn* CNVs

The Mammalian Phenotype Ontology, the set of terms by which the MGI annotates the phenotypes of mouse models, is organised at its highest level into 30 over-arching phenotypes [21]. Of these, three categories (*Behavior/Neurological*, *Hearing/Vestibular/Ear* and *Lethality-Postnatal*) were significantly over-represented by AGP ASD *dn* CNV genes compared to expectation by random chance, thus associated with AGP ASD *dn* CNV genes (BH-adjusted one-sided Fisher's test *p*<5%; Table S3). Importantly, these significant associations are all specific to *Gain* CNVs (2.0–2.7-fold increases) whilst observed counts for *Loss* CNVs differ little from expected values (data not shown).

**Figure 1:**
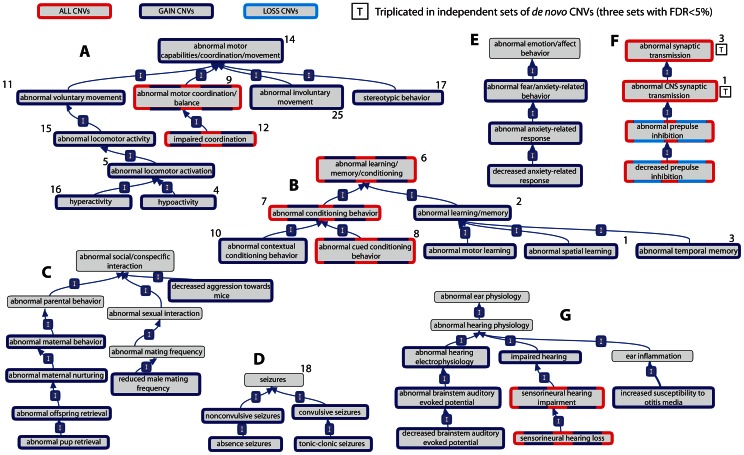
Relationships of mouse model phenotypic terms enriched among genes overlapped by *de novo* CNVs identified in individuals with ASD. Relationships between phenotypic terms within the Mammalian Phenotype Ontology are indicated by a blue arrow running from the child term to the parent term. Terms are significant (BH-adjusted *p*<5%) in at least one of 4 sets of *de novo* CNVs identified in individuals with autism if they are shown with a coloured border (red, dark and light blue). Those terms whose significant enrichment is observed in three independent sets, and thus triplicated, are marked with a boxed letter “T”. Panels A–E show representative clusters of *Behaviour/Neurological* phenotypic category, while Panel F shows the enriched phenotypes from the *Nervous System* phenotypic category and Panel G shows representative enrichments from the *Hearing/Vestibular/Ear* phenotypic category. The number adjacent to the phenotypic terms indicates the rank of that phenotypic term among those phenotypes significantly enriched among a set of 22 disease genes previously implicated in ASD (see Results).

The significant enrichments of these three overarching categories with the AGP ASD *dn* CNVs then provided the rationale necessary for testing of all their finer-scale phenotypic terms for association (162, 218 and 2 terms, for *Behaviour/Neurological*, *Hearing/Vestibular/Ear* and *Lethality-Postnatal* categories, respectively; see Materials and Methods). Although the *Nervous System* phenotypic category was not significantly over-represented among ASD *dn* CNV genes (*All* AGP ASD *dn* CNVs: 1.3-fold increase, *p* = 0.03, BH-adjusted *p*>5%), the behavioural presentations of ASD are likely to be manifestations of nervous system abnormalities. Consequently, we also tested for significant enrichments of finer-scale phenotypes within this category (282 terms). Subsequently, 23 behavioural, 21 nervous system, 27 hearing and 1 postnatal-lethality phenotypes were identified as being significantly enriched among the AGP ASD *dn* CNV genes (BH-adjusted *p*<5%; Figure 1 and Table S3). For 3 CNV sets, namely AGP, Marshall *et al.* and Levy *et al.*, we also identified a significant excess of genes associated with *abnormal CNS synaptic transmission* phenotypes in mice, thereby triplicating this association (AGP 3.0-fold enrichment, *p* = 7×10^−5^, BH-adjusted *p*<5%; Marshall *et al.* 2.1-fold enrichment, *p* = 1×10^−4^, BH-adjusted *p*<5%; Levy *et al.* AGP 2.2-fold enrichment, *p* = 5×10^−5^, BH-adjusted *p*<5%; Sanders *et al.* 1.6-fold enrichment, *p* = 0.008, BH-adjusted *p*>5%; Table 1, Table S3).

**Table 1:** Triplicated mouse model phenotype associations among genes overlapped by sets of *de novo* CNVs identified in individuals with ASD.

MGI mouse model phenotype	Mammalian Phenotype Ontology identifier	Phenotype definition	ASD *de novo* CNV sets significantly enriched	Enrichment (rank within category) among *AGP-Implicated* genes	Enrichment among *AGP control* CNVs
*Abnormal Synaptic Transmission*	MP:0003635	*Defect in the communication from a neuron to a target across a synapse*	AGP-*All* (2.5-fold), Marshall-*All* (2-fold), Levy-*All* (2.1-fold)	5.5-fold (3)	1.2-fold
*Abnormal CNS synaptic transmission*	MP:0002206	*Defect in the communication from a neuron to a target across a synapse in the central nervous system*	AGP-*All* (3-fold), Marshall-*All* (2.1-fold), Levy-*All* (2.2-fold)	6.5-fold (1)	1.3-fold

Enrichments are given as the fold change over that expected by chance (see Materials and Methods).

### Genes previously implicated in ASD are also associated with these model phenotypes

We next sought to determine whether model phenotypes enriched among the mouse orthologues of genes previously implicated in ASD are equivalent to those we now associate with ASD *dn* CNVs. Of 36 genes that had been causally-implicated in ASD by previous studies, as defined previously [6], phenotypic annotations were available for the unique mouse orthologues of 26 (see Materials and Methods). We removed 4 genes that were also overlapped by an ASD *dn* CNV to form a wholly independent set of 22 genes herein termed *ASD-Implicated* genes (Table S4).

We observed a striking concordance between the model phenotypes associated with the *ASD-Implicated* genes and those associated with the ASD *dn* CNV genes despite these sets' complete independence: the two abnormal synaptic phenotypes with triplicated associations to ASD *dn* CNVs ranked 1^st^ and 3^rd^ among those *Nervous System*-category phenotypes that were most significantly associated with *ASD-Implicated* genes, while 15 of the top 18 *behavioural*-category phenotypic associations among *ASD-Implicated* genes were among those independently associated with the AGP *dn* CNVs (Figure 1, Table 1, Table S4).

### The protein products of genes that contribute to these phenotypic associations interact

**Figure 2:**
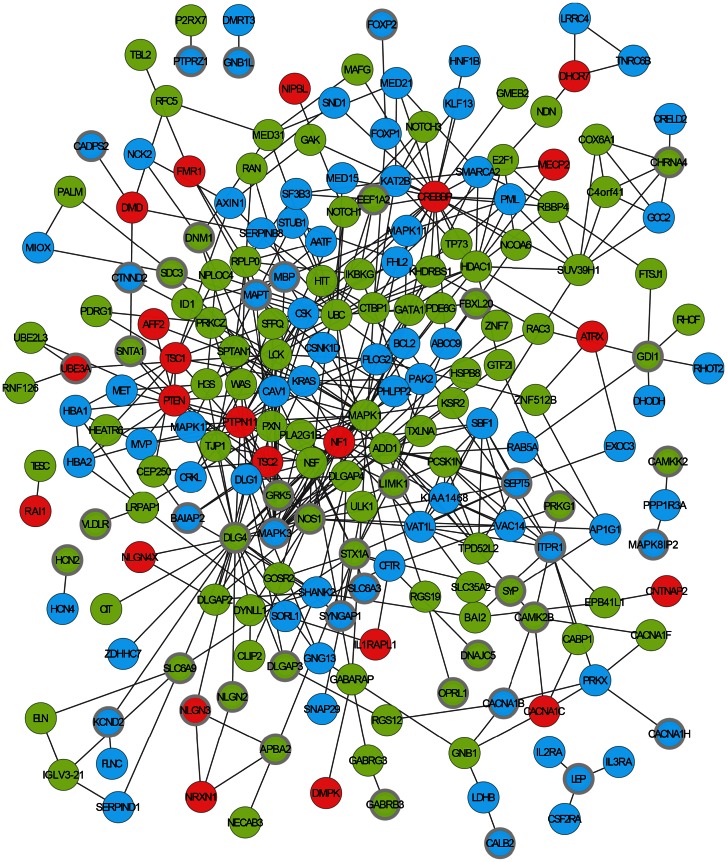
An *ASD-associated* interaction network. The network is formed from direct protein-protein interactions between the products of ASD *dn* CNV genes that are associated with *synaptic* phenotypes (shown with thicker grey border), genes previously implicated in ASD (*ASD-implicated* genes), and other ASD *dn* CNV genes whose products directly interact with these gene's products. Physical interactions between two proteins are shown as an edge connecting two circles representing each gene. Genes found to be duplicated in autistic patients in this study are shown in green, deleted genes in blue, and *ASD-implicated* genes in red. An alternative and more detailed view of this network is shown in Figure S6.

Given the repeated enrichment within independent CNV sets of genes whose mouse orthologues are associated with *abnormal synaptic transmission* phenotypes, we asked whether the protein products of the 59 *synaptic* phenotype CNV genes taken from across all sets might interact within common processes or pathways. Even after correcting for the increased likelihood that the products of genes with behavioural or neurological associations interact, our analysis showed that the number of these proteins' interactions is unexpectedly high (3.75-fold over-representation, *p* = 0.006; Figure 2, Table 2 and Table S5; see Materials and Methods). When we then added the set of 36 *ASD-Implicated* genes, the number of direct protein interactions increased yet further (3.2-fold over-representation, *p*<0.002). Cumulatively, our results show that many of the 59 *synaptic* phenotype CNV genes and 36 *ASD-Implicated* genes function in concert and yield similar consequences when disrupted (Figure 2, Table 2).

**Table 2:** Protein-Protein Interaction (PPI) network enrichments.

Gene Set	Genes	Direct PPIs	*P*-value	Enrichment
*Abnormal Synaptic Transmission (AST)* ASD *dn* CNV genes	59	22	0.006	3.8-fold
*AST* ASD *dn* CNV genes + *ASD-Implicated* genes	90	43	<0.002	3.2-fold
*AST* ASD *dn* CNV genes + *ASD-Implicated* genes+other directly-interacting ASD *dn* CNV genes	233	452	<0.002	5.4-fold

Enrichments are given as the fold change over that expected by chance (see Materials and Methods).

Mouse model phenotypic information is available only for the orthologues of fewer than a quarter of human genes (see Materials and Methods). It is thus expected that not all genes within CNVs that are causally associated with synaptic abnormalities can be identified using this resource alone. To identify additional ASD candidate genes, we sought all those ASD *dn* CNV genes whose protein products were known to directly interact with the products of any of the 59 *synaptic* phenotype CNV genes or 36 *ASD-Implicated* genes. This identified an additional 174 CNV genes that form an expanded network with a 5.4-fold interaction over-representation (*p*<0.002; herein termed the *ASD-associated* network; Figure 2, Table S5). Of these 174 additional interacting proteins, the mouse orthologues of 74 (43%) do not yet have phenotypic information. Of the 100 additional interacting genes with mouse model phenotypes, 44 are known to exhibit behavioural phenotypes, and of these 35 exhibit one or more of the significantly associated behavioural phenotypes identified above (Figure 1 and Table S5). Examining the more general functional annotations of genes within the *ASD-associated* network using Gene Ontology (GO) identifies convergent functional themes that are consistent with broad synaptic functioning, organisation and maintenance (Table S6; Summarised using REVIGO in Figures S1, S2 and S3 [22]). This functional coherence is supported by the observation that 192 of the 210 (91%) proteins within the *ASD-associated* network reside in a single inter-connected cluster, thereby also providing known interactions that provide pathways through which effects originating from distinct mutations can aetiologically converge (Figure 2). Despite their known functional interconnections, the vast majority of these ASD candidate genes are novel (Table S5).

The 203 CNV genes singled-out through the *synaptic* mouse phenotype associations and the *ASD-associated* network provide a causal hypothesis for 81 (45%) of the patients considered. The median number of candidate genes per patient is 3 (mean 3.8, s.d. 3.2) suggesting a substantial role for epistasis in ASD. The network identified here provides not only the means for mediating epistatic interactions but is also indicative of the deleteriousness of copy change: Among the 22 patients that have only a single copy-changed candidate gene, that candidate gene has on average 3 times the number of interaction partners in the network as compared to the candidate genes from patients with multiple candidate genes (medians 3 vs. 1, respectively, Mann-Whitney U test *p* = 0.002). Thus, given the known deleteriousness of disrupting highly interacting “hub” genes within biological networks [23], we propose that the disruption of multiple non-hub genes within the autism network may be required to elicit an autistic phenotype comparable to the singular disruption of a hub gene within the same network.

### Duplicated and deleted ASD candidate genes converge on common aetiologies

**Figure 3:**
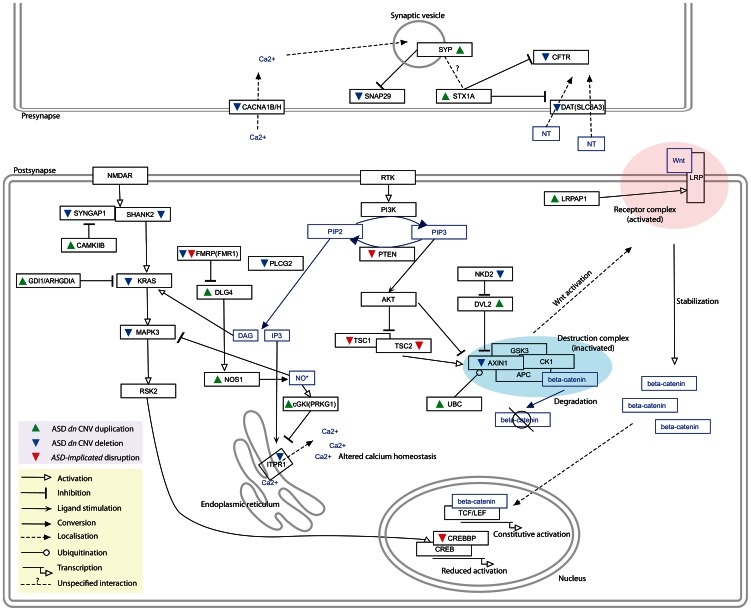
Distinct duplications and deletions of genes whose proteins interact within the *ASD-associated* network perturb pathways in the same direction. Genes duplicated within ASD *dn* CNVs are indicated with green upwards arrows while those deleted are denoted by blue downwards arrows. Previously identified *ASD-Implicated* genes found to be disrupted in autism patients are denoted with red downwards arrows. The nature of the interactions/regulations between proteins/molecules are shown with different edge types (see in-figure legend). The *ASD-associated* network (Figure 2) identifies several deletion/duplication pathway cascades, for example the *MAPK3* pathway (see Discussion for additional examples). Here, deletions of the MAPK3 pathway components (i.e. *SYNGAP1*, *SHANK2*, *KRAS*, *MAPK3*, *PAK2*, and *CREBBP*) and duplications of their negative regulators (i.e. *FMR1*, *GDI1*, *ARHGDIA*, *CAMK2B*, and *CAMKK2*) found in autistic patients identify converging effects on the MAPK pathway, specifically reduced CREB-dependent transcription [9], [62], [63], [64]. CREB-dependent transcription has been implicated in neuroadaptation [20]. In addition, increased NO* production leads to the inhibition of *MAPK1/3* activity [65], which fits well with the observed CNV duplications of both *NOS1* and *DLG4*, the latter gene promoting recruitment of *NOS1* [66]. Similarly, duplication of *PRKG1*, which is up-regulated by NO* and expresses a product that inhibits IP3 production, is predicted to reduced activation of the calcium-releasing IP3-receptor *ITPR1* [67], which is in turn found to be deleted.

Of the 203 CNV genes identified through the *synaptic* mouse phenotype associations and the *ASD-associated* network, 110 (54%) are found only in duplications while 91 (45%) are only in deletions. We next investigated how the two directions – duplications or deletions – of copy number change might reflect common or divergent aetiologies. To achieve this we analysed the GO *biological process* annotations assigned to duplicated and, separately, to deleted genes for significantly over-represented terms (Table S6). While many of the over-represented annotation terms are shared between the deleted and duplicated gene sets, we noted a striking difference: The deleted candidate genes are significantly enriched only in genes that are positive regulators of biological processes (GO:0048518, 35/82 annotated genes, 2.4-fold enrichment, BH-adjusted *p* = 3×10^−4^) while, conversely, an enrichment of genes that are negative regulators of biological processes is only observed amongst the duplicated candidate gene set (GO:0048519, 34/105 annotated genes, 2-fold enrichment, BH-adjusted *p* = 0.006; Table 3). Each of the 4 CNV set's candidate genes contribute to each of these enrichments with many sets nominally significant individually (Table S7). Furthermore, reclassifying the partially duplicated, and therefore likely-disrupted, genes as deletions enhances these enrichments further (Table S8). These enrichments are complementary and thus immediately suggest a convergent model of action in which the duplication of negative regulator genes or the deletion of positive regulator genes both act to perturb a common target process and affect the same outcome. The unusually frequent deletions of positive regulators and duplications of negative regulators enable specific and biologically-meaningful interpretations of the *ASD-associated* network (see Figure 3 and Discussion).

**Table 3:** Regulatory GO enrichments amongst ASD *dn* CNVs candidate genes.

Copy Change Direction	Total GO genes	Positive regulators (GO:0048518)			Negative Regulators (GO:0048519)		
		Genes	BH-adjusted *p*-value	Enrichment	Genes	BH-adjusted *p*-value	Enrichment
*Duplications*	105	24 (23%)	>1	1.3-fold	34 (32%)	**0.006**	2.0-fold
*Deletions*	82	35 (43%)	**0.0003**	2.4-fold	24 (29%)	0.08	1.8-fold

The set of candidate genes was defined as those CNV genes associated with *Abnormal Synaptic Transmission* mouse phenotypes (Table 1) and those CNV genes identified through direct protein-protein interactions (Table 2). Enrichments are given as the fold change over that expected by chance (see Materials and Methods).

## Discussion

This study has (*i*) identified among 3 independent sets of ASD *dn* CNVs, and therefore triplicated, an enrichment of genes whose mouse orthologues, when disrupted, yield an *abnormal synaptic transmission* phenotype; (*ii*) shown that these genes' protein products exhibit a significantly high number of interactions between themselves and to the products of genes previously implicated in ASD; (*iii*) that this interaction network extends directly to include many more proteins of genes affected by the ASD *dn* CNVs of almost half of the cohort; (*iv*) that the gene products in this *ASD-associated* network possess roles in synaptic function, organisation and maintenance; (*v*) that many individuals with ASD possess multiple copy changed genes from the *ASD-associated* network; (*vi*) that genes that are highly connected within the network (“hub genes”) are significantly enriched among patients that possess only a single *ASD-associated* network gene; and, finally (*vii*) that this network's genes that are deleted are significantly enriched in genes that act to positively regulate biological processes while those that are duplicated are significantly enriched in negative regulators.

An association of ASD CNVs with genes that yield synaptic phenotypes when disrupted in mice has been reported before in rare CNVs but replication was not achieved [13]. Here, despite little overlap between the 3 CNV sets involved (Table S2E), we are able to triplicate this association. These synaptic associations provide aetiological insight that accords well with the emerging neurophysiological view of a strong role for synaptic dysfunction in autism [24]. It is also further strengthened by the over-represented functions among genes within the broader *ASD-associated* network, whose functions include vesicle transport, cell junction organisation and calcium transport (Figures S1, S2 and S3, Table S6). However, the breadth of dysfunction suggested by the roles of these physically-interacting proteins implicate other, more intracellular processes, such as the cytoskeletal and cellular transport processes, that may affect synapse formation, structure and/or maintenance (Figure S1, Table S6).

The known physical interactions between these genes' products provide pathways through which separate genetic perturbations can converge functionally (Figure 2, Table S5) while the importance of a gene within the *ASD-associated* network, as specified by the degree of connectivity, appears to be an indicator of ASD-relevant deleteriousness (see Results). Recently, two large-scale studies examining the exomes of autistic patients also identified an excess of protein-protein interactions between genes harbouring suspected causative mutations, reporting smaller networks with 49 [15] and 45 [14] participating genes of which 3 genes and 2 genes, respectively, are also identified through our network. After excluding overlapping genes and compared to random gene sets of equivalent size, the number of connections between gene products in each of the O'Roak *et al.* and Neale *et al.* reported networks to the network we identify here are 12-fold and 38-fold over-represented, respectively (*p*<0.002 for both). Thus, despite little overlap in genes, the strong interconnectedness between these networks identifies pathways through which cellular perturbations arising from distinct mutations identified in separate studies may converge. The single nucleotide variants (SNVs) detected in these two published exome studies are largely predicted to be harmful to the function of the encoded proteins, and therefore comparable to the copy number deletion events in our study. Corroborating our finding of an enrichment of genes that positively regulate among deletions, we also observe a highly significant enrichment of positive regulators among the more strongly-interconnected set of genes identified by Neale *et al.* (2.7-fold enrichment, *p* = 3.8×10^−6^, BH-adjusted *p*<0.05) which, while enriched, is not significant among the less well-connected genes reported by O'Roak *et al.* (1.4-fold enrichment, *p*>0.05).

Despite chronologically limiting our mouse phenotypic dataset to avoid bias (see Materials and Methods), the similarities between the behavioural mouse phenotypes associated with the AGP *dn* CNVs and human ASD presentations appear clear, with *abnormal social/conspecific interaction*, *stereotypic behaviour* and *abnormal memory/learning/conditioning* phenotypes all over-represented (Figure 1). Many of our study's ASD-associated phenotypes bear a striking resemblance to other frequently co-occurring symptoms, such as *impaired coordination* [25], [26], [27], anxiety-related phenotypes [28], and *absence* and *tonic-clonic seizures* [29], [30], [31] (Figure 1). Finally, we observe a strong enrichment of genes whose disruption yields hearing phenotypes in mice. This observation accords well with estimates in the literature that hearing abnormalities (including sensorineural hearing disorders) affect between 33–46% of ASD cases (Figure 1G) [32], [33]. Many of the associated hearing, and some nervous system, mouse phenotypes are related to peripheral hearing abnormalities, particularly concerning the cochlea and mechanoreception (Figure S4 and Table S3). Inner ear mechanoreception abnormalities appear to have received little attention compared to other regions involved in auditory reception and processing [33]. This is despite improvements in hearing following cochlear implants in individuals with ASD [34] and the knowledge that rare mutations in several genes implicated in ASD (including *CHD7*, *NIPBL*, *PTPN11* and *TBX1*) can cause inner ear abnormalities in humans [35], [36], [37], [38].

The enrichment of deleted genes in the network that positively regulate biological processes and a complementary enrichment of duplicated genes that negatively regulate biological processes suggest the occurrence of convergent aetiologies whereby both deletions and duplication act to perturb biological processes relevant to autism in the same direction (Figures 2 and 3). Indeed, the interactions within the *ASD-associated* network reveal this proposition to be consistent with the experimental literature. For example, considering the STX1A/CFTR/SLC6A3 (aka. DAT) interactions (Figure 3), over-expression of *STX1A* decreases DAT dopamine transport activity [39], and reduces the CFTR-mediated chloride current by inhibiting trafficking of CFTR to the cell surface [40]. These findings predict that over-expression of *STX1A* yields an effect similar to the deletion of *DAT* or *CFTR* and, concordantly, whereas *STX1A* lies within a duplication, *CFTR* and *DAT* are each deleted. Furthermore, STX1A also interacts with SYP (also duplicated), which negatively regulates SNAP proteins (*SNAP29* is deleted) [41], [42]. SNAP proteins are key to presynaptic exocytosis, a process also likely to be disturbed by altered calcium homeostasis resulting from the array of deleted voltage-dependent calcium channels (CACNA1B [deletion], CACNA1C [*ASD-implicated* deletion], and CACNA1H [deletion]) [43] (Figure 3).

Another example of apparent convergence in aetiology and outcome are the copy number changes affecting the PI3K/*Wnt* pathways (Figure 3). Here, many copy number changes are predicted to converge to reduce or disrupt the action of the *β-catenin* destruction complex in the *Wnt/β-catenin* signalling pathway; the deletion of *AXIN1*, the increased ubiquitination of AXIN1 by duplication of UBC [44] or the disruption of the PI3K pathway due to mutations in *PTEN*, *TSC1*, or *TSC2* [45] (Figure 3). Perturbations affecting AXIN1in ASD include the duplication of *DVL2* which inhibits *AXIN1* (deleted) function [46]. Furthermore, as NKD family proteins promote the degradation of DVL proteins [47], [48], the deletion of *NKD2* may increase the activities of *DVL2* and thereby also inhibit AXIN1. Concordant with a decrease in *β-catenin* degradation, an increase in *β-catenin* stabilization could result from *LRPAP1*, *NKD2* and *DVL2* copy number changes. LRPAP1 is thought to have protective roles in LRP1 trafficking and its duplication may therefore increase LRP1 availability [49]. The outcome of the copy number change and disruption of each of these genes is likely to up-regulate the *Wnt*-stimulated TCF/LEF-dependent transcription, a pathway whose down-regulation has been proposed to have therapeutic benefits in ASD models [50], [51], [52].

Given the ever-increasing number of genetic variants that thus far have been implicated in ASD, the focus will inevitably shift from enumeration towards understanding how these variants contribute to the common pathways and processes underlying this complex disease. Here we have identified a large network of interacting proteins affected by copy number variants identified in patients with ASD, and shown how the network topology and direction of copy number change can be used to interpret these variants' pathway perturbations. Therapeutically targeting molecules at the ends of pathologically-perturbed regulatory cascades may provide more broadly-applicable treatments, while pathological gene duplications may identify attractive targets for knock-down therapeutics as a means of ameliorating perturbed pathways.

## Materials and Methods

### *De novo* CNVs identified in individuals with ASD

Four sets of *de novo* CNVs were employed in this study (Table S1), of which two are drawn from the Simons Simplex Collection and thus overlap [53]. The largest set consists of 73 *de novo* CNVs identified in 54 (out of 996) individuals with strict autism by the Autism Genome Project (AGP; Table S1) [6]. Of these, 39 CNVs had been confirmed as *de novo* by independent methods while 34 were considered likely to be *de novo* by the CNV calling algorithms. The second set consisted of 28 *de novo* CNVs identified in 24 patients reported in a study by Marshall *et al.* [54]; two patients who had been reanalysed by the AGP have been removed; Table S2. The third set consisted of 94 *de novo* CNVs identified in 82 patients reported in a study by Levy *et al.* [20] and the fourth set consisted of 67 *de novo* CNVs identified in 63 patients reported in a study by Sanders *et al.* [55]. Forty two patients examined by Levy *et al.* were also present in the study by Sanders *et al.* but this does not affect our findings; As the synaptic phenotype associations that we report and take forward in the Results were identified amongst both the AGP and the Marshall *et al.* sets, neither the Levy *et al.* nor Sanders *et al.* sets were required for replication and thus these latter sets non-independence from each other does not undermine this association. For all sets, contributing patients have been evaluated as having ASD according to ADI-R and/or ADOS criteria. Herein, *de novo* CNVs identified in patients with ASD is termed “ASD *dn* CNVs”.

### Assigning genes to CNVs

Human genes were assigned to ASD *dn* CNVs according to Ensembl Ensmart54 [56]. To be confident that the expressed coding sequence of a gene is affected by the copy number change, we conservatively required at least one coding exon of every known transcript of a gene to be overlapped by a CNV for that gene to be deemed overlapped (Table S2). Particular consideration was given to showing that our gene assignment procedure and statistical over-representation analyses did not yield any functional bias under the null hypothesis (see Figure S5 and Methods S1). Genes observed to be copy number variable in the same direction (gain/loss) within a set of CNVs employed by the AGP as a control, i.e. identified from individuals with no obvious psychiatric history in a previous study were removed from the ASD *dn* CNV gene lists because these are less likely to be associated with ASD [6]. Although it remains possible that common variants contribute to ASDs, our study focuses on genes affected by rare, *de novo* variants (see Introduction).

### Mouse Genome Informatics (MGI) phenotypes

Annotations of phenotypes resulting from disruptions of mouse orthologues of these affected genes were obtained from the Mouse Genome Informatics (MGI) resource (http://www.informatics.jax.org) and interpolated as described previously [12], [57], [58], [59]. Using simple, unambiguous, 1∶1 gene orthology relationships from the MGI resource, 5,283 distinct MGI phenotypic terms were mapped to 5,671 human genes. Each phenotype belongs to one or more of 33 over-arching categories. We considered only 4,055 reasonably populated phenotypes, defined as those with at least 1% of all genes associated with the relevant phenotypic category, thereby reducing uninformative results and improving methodological power. As an unreplicated association between genetic variants in autism patients and a mouse model phenotype was reported in April 2010 [13], we employed only those phenotypes reported in the MGI resource prior to this date, thereby reducing any subsequent phenotyping bias or consequential circularity in discovery. However, our findings remain, or are strengthened by, those more recently reported mouse model phenotypes (data not shown).

### Protein-protein interactions

We employed DAPPLE: Disease Association Protein-Protein Link Evaluator [60] to identify direct protein-protein interactions among the protein products of the genes contributing to functional enrichments. A protein-protein interaction network's connectivity was calculated as published previously [60]. Enrichment analysis was carried out by comparing the number of identified direct protein interactions with the average of those identified from 500 gene sets, in which genes were randomly sampled while matched in set size. To account for the increased likelihood that genes that share behavioural associations are more likely to interact than randomly selected genes, we randomly selected sets of orthologues from 1,766 genes annotated with behaviour and neurological phenotypes in the MGI.

### Statistical analysis

Due to the small numbers of *de novo* CNVs considered here and a lack of a control set of *de novo* CNVs, performing a case-control comparison is not possible. Thus, employing the one-sided Fisher's exact test, we tested the null hypothesis that a (mouse) phenotype associated with (human) Ensembl genes overlapping a set of ASD-associated CNV genomic intervals occurs at a frequency that is no different from that expected from the genome as a whole. Randomisations confirmed that this approach did not yield artefactual bias (see Methods S1 and Figure S5). A multiple testing correction, BH-adjusted *p*<5%, was applied to account for number of functional terms (phenotypes or GO terms) tested when examining a given gene set [61].

## Supporting Information

Figure S1 REVIGO-summarised Biological Process Gene Ontology terms enriched within the combined set of ASD *dn* CNV genes that are associated either with an *abnormal synaptic transmission* phenotype in the mouse or whose protein product directly interacts with an ASD *dn* CNV gene that is associated with this phenotype (Supek et al., 2011; The Gene Ontology Consortium et al., 2000). The summarised GO terms are given in full and separately for deletions and duplications in Table S6.
(TIFF)

Figure S2 REVIGO-summarised Cellular Location Gene Ontology terms enriched within the combined set of ASD *dn* CNV genes that are associated either with an *abnormal synaptic transmission* phenotype in the mouse or whose protein product directly interacts with an ASD *dn* CNV gene that is associated with this phenotype (Supek et al., 2011; The Gene Ontology Consortium et al., 2000). The summarised GO terms are given in full and separately for deletions and duplications in Table S6.
(TIFF)

Figure S3 REVIGO-summarised Molecular Function Gene Ontology terms enriched within the combined set of ASD *dn* CNV genes that are associated either with an *abnormal synaptic transmission* phenotype in the mouse or whose protein product directly interacts with an ASD *dn* CNV gene that is associated with this phenotype (Supek et al., 2011; The Gene Ontology Consortium et al., 2000). The summarised GO terms are given in full and separately for deletions and duplications in Table S6.
(TIFF)

Figure S4 Enriched cochlear and mechanoreception-associated mouse model phenotypes among orthologues of genes overlapped by *Gain de novo* CNVs from the AGP set. Panel A shows the relevant terms that were identified among *Nervous System* category phenotypes while Panel B shows the relevant terms that were identified among *Hearing/Vestibular/Ear* category phenotypes. Relationships between phenotypic terms within the Mammalian Phenotype Ontology are indicated by a blue arrow running from the child term to the parent term. Terms are significantly enriched (BH-adjusted *p*<5%) if they are shown with a blue border.
(TIFF)

Figure S5 Distributions of *p*-values for the surfeits of genes associated with 3 phenotypic categories obtained for 500 randomised sets of case-matched CNVRs. 500 sets of genomic regions matched in size and number to each of the *Loss* and the *Gain* CNVRs were obtained (see Materials and Methods). For each set, the likelihoods for the observed surfeit of genes associated with a particular phenotypic category, namely *Behavior[/Neurological]*, *Nervous System* and *Hearing/Vestibular/Ear*, were recorded and a histogram charted. See also Methods S1.
(TIFF)

Figure S6 An alternative view of the ASD-associated interaction network shown also in Figure 2 **.** The network is formed from direct protein-protein interactions between the products of ASD *dn* CNV genes that are associated with synaptic phenotypes (shown in with thicker grey border), genes previously implicated in ASD (*ASD-implicated* genes), and other ASD *dn* CNV genes whose products directly interact with these gene's products. Physical interactions between two proteins are shown as an edge connecting two circles representing each gene. Genes found to be duplicated in autistic patients in this study are shown in green, deleted genes in blue, and *ASD-implicated* genes in red.
(TIFF)

Methods S1 Statistical analysis and gene assignment. Supporting materials for our method of gene assignment and confirming the null of statistical analysis. See also Figure S5.
(DOC)

Table S1 Genomic extent and ENSEMBL gene content for ASD-associated *de novo* CNVs and AGP control CNVs. In CNV datasets, the genes considered are those remaining after excluding genes also overlapped by benign CNVs in the same direction of copy change (see Materials and Methods). Sets termed All are formed by combining both Gain and Loss CNVs.
(XLSX)

Table S2 (5 sheets): *De novo* CNVs identified in individuals with ASD employed in this study: Sheet A lists the *de novo* CNVs reported by the Autism Genome Project (AGP), Sheet B lists the *de novo* CNVs reported in the Marshall *et al.* study, Sheet C lists the *de novo* CNVs reported in the Levy *et al* study and Sheet D lists the *de novo* CNVs reported in the Sanders *et al* study. Sheets A–D provide the genomic location (NCBI36) and copy change for the AGP set of CNVs employed in this study. Sheet E details the overlapping sequence between the three sets of *de novo* CNVs considered in the study. The total amount of sequence covered by each set is given next to the set name in the row headings. The percentage shown in brackets in the main body gives the proportion of the set listed in the row heading that is overlapped by the set listed in the column headings.
(XLS)

Table S3 (11 sheets): Significantly enriched mouse model phenotypes among the orthologues of genes overlapped by 3 independent sets of *de novo* CNVs identified in individuals with ASD: Sheets (A–E) AGP *de novo* CNVenrichments, sheets (F–H) Marshall *et al. de novo* CNV enrichments, and sheets (I–K) Levy *et al. de novo* CNV enrichments. For AGP enrichments (sheets A–E), the attached excel spreadsheet provides 5 spreadsheets/tables: one sheet/table each for enrichments in three phenotypic categories *Behaviour/Neurological* (sheet A), *Hearing/Vestibular/Ear* (sheet B) and *Nervous System* (sheet C); The AGP candidate genes identified through these enrichments (sheet D); and the AGP CNVs for which candidate genes have been identified (sheet E). For the Marshall *et al.* enrichments, 3 spreadsheets/tables are provided: the enrichments in the phenotypic category *Nervous system* (sheet F); The Marshall *et al.* candidate genes identified through these enrichments (sheet G); and the Marshall *et al.* CNVs for which candidate genes have been identified (sheet H). For the Levy *et al.* enrichments, 3 spreadsheets/tables are provided: the enrichments in the phenotypic category *Nervous system* (sheet I); The Levy *et al.* candidate genes identified through these enrichments (sheet J); and the Levy *et al.* CNVs for which candidate genes have been identified (sheet K). Phenotype tables: PhenotypeID is the Mammalian Phenotype Ontology identifier. PhenotypeName (Abbreviation: Total Genes in Genome with Phenotype) provides the phenotype name. Within the brackets, the phenotype abbreviation for use in other tables is given first, while the second number gives the total number of human orthologues with a mouse orthologue that yields this phenotype when disrupted. CopyChangeSet = ALL|GAIN|LOSS gives the direction of copy change of the CNVs whose overlapping genes possess this enrichment. Percentage Increase is the enrichment of genes associated with this phenotype over that expected from the genome by chance (see Materials and Methods). *P*-value is the associated probability for this enrichment (hypergeometric probability, BH-adjusted *p*<5%). Number of Genes forming Enrichment is the number of overlapped genes that contribute to this enrichment. Total Genes in CNV Set gives the number of genes with this CNV set for which phenotypic information is available. Number of ASD implicated genes hit by enrichment gives the number of genes within this enrichment that also belong to a set of 26 genes that have both been previously implicated in ASD as defined by the AGP Consortium and for which phenotypic information is available. Number of CNV hits (unique CNVs, percentage of CNVs) gives the total number of genes from this enrichment that overlap a CNV within this set. In parenthesis, the first number is the number of unique CNVs from this set that are overlapped by at least one gene from this enrichment, while the second number gives the proportion of all CNVs within this set that are overlapped. Number of patients hits (unique patients, percentage of patients) gives the total number of genes from this enrichment that overlap a *de novo* CNV within a patient from this cohort. In brackets, the first number is the number of unique patients from this set that possess a *de novo* CNV that is overlapped by at least one gene from this enrichment, while the second number gives the proportion of all patients within this cohort that possess a CNV that is overlapped. Genes hit lists the genes that contribute to this enrichment. Ensgs hit gives the ENSEMBL ids for the contributing genes. CNVs hit gives the CNVs that are overlapped by genes from this enrichment. The CNV format is [cnv_identifier]_chr[chromosome]_[gain/loss]_[chromosome start position]. Candidate Gene Table: GENE is the gene name, ENSG is the ENSEMBL identifier, Phenotypic enrichments contributed to lists the enriched phenotypes for this set to which this gene contributes. The phenotypic abbreviations are listed in the phenotypic enrichment table(s) for this CNV set in the previous spreadsheet/table within this excel workbook. Number of CNVs gives the number of CNVs in this set that candidate gene overlaps. CNVs lists the particular CNVs overlapping this gene (see phenotype sheet/table for format description). Patients lists the patients possessing these CNVs. CNVs hit Table: CNV gives the CNV from this set that is overlapped by a candidate gene (see phenotype sheet/table for format description). Number of Candidate Genes hit is the number of genes that contribute to the significant enrichments observed for this set of CNVs that are overlapped by this particular CNV. Genes (enriched phenotypes) lists the overlapped candidate genes and gives abbreviations for the enriched phenotypes that this gene contributes to - see phenotype tables/sheets for abbreviation listings.
(XLS)

Table S4 (5 sheets): Significantly enriched mouse model phenotypes among the orthologues of 22 genes that have been implicated in ASD as defined by the AGP Consortium. (A) ASD Implicated Genes lists twenty two human genes deemed previously to have been implicated in ASD (termed *ASD-Implicated* genes). These genes are a subset of 36 disease genes deemed causally implicated in ASD by the Autism Genome Project (Pinto et al., 2010), and was formed by retaining only those genes whose mouse orthologue's disruption had been phenotyped and discarding those genes that were overlapped by ASD *dn* CNVs considered in this study. (B) Categories lists the enriched Mammalian Phenotype Ontology categories: (**C**) Nervous System, (D) Behavior/Neurological, (E) Lethality-Postnatal each list the finer phenotypes enriched within each of these categories respectively. The probability is that obtained through the hypergeometric test (BH-adjusted *p*<5%). Total Genes with Phenotype gives the total number of human genes whose mouse orthologue is associated with this phenotype. Set Genes with Phenotype is the total number of genes within the set whose mouse orthologue is associated with this phenotype. Percentage change over expected is that obtained when compared with the genome as whole sampled randomly.
(XLS)

Table S5 (6 sheets): Candidate ASD CNV genes identified in this study. (A) All CNV candidate genes are listed, along with the ASD *dn* CNVs that harbour them. PPI partner(s) lists other candidate genes whose proteins directly interact with the protein product of the listed gene. (B) 59 ASD *dn* CNV genes whose mouse orthologues' disruption yield a synaptic phenotype. (C) 144 ASD *dn* CNV genes whose protein products interact with the protein products of either the ASD *dn* CNV genes whose mouse orthologues' disruption yield a synaptic phenotype listed in sheet B or the 22 *ASD-Implicated* genes listed in Table S4. (D) Each ASD *dn* CNV with a candidate gene is listed along with the candidate genes proposed by this study. Total Genes Considered lists the number of protein-coding genes within that CNV. (E) Each patient with autism for whom a candidate gene is proposed in this study is listed. (F) 1∶1 PPI partners and DAPPLE network parameters.
(XLS)

Table S6 Gene Ontology terms significantly enriched among 204 candidate genes identified in this study. Probability is the associated *P*-value for this enrichment (hypergeometric test, BH-adjusted *p*<5%). Total Genes in Genome with annotation gives the total number of human genes annotated with this GO term. Sample Genes with annotation is the total number genes within the set annotated with this GO term. % difference over expected is that obtained when compared with the genome when sampled randomly.
(XLS)

Table S7 Genes annotated with GO terms “positive regulation of biological process” (GO:0048518) and/or “negative regulation of biological process” (GO:0048519) within each set of *de novo* CNVs considered in this study. The *p*-value is the raw uncorrected value derived from comparison to all genes. “%Change” gives the percent change over or under that expected against the background of all genes.
(XLSX)

Table S8 Regulatory GO enrichments amongst ASD dn CNVs candidate genes after reclassifying partially-duplicated genes as deleted. The candidate genes considered here are the subset of CNV genes whose orthologues have synaptic phenotypes in mice plus other CNV genes whose protein products directly interact with the synaptic genes' proteins. If the outcomes of many of these genes' duplications result in the increased down-regulation of their targets, then each such gene would be expected to be duplicated largely in its entirety for the duplication to be functional. Indeed, after removing partially-duplicated genes, we find that the enrichment of duplicated genes that negatively regulate biological processes increases (32/92 genes, +119% enrichment, BH-adjusted *p* = 0.005; Table 1). Concomitantly, including the partially-duplicated genes that would be expected to functionally resemble deletions, with the deleted genes maintains the enrichment of positive regulators (40/95 genes, +134% enrichment, BH-adjusted *p* = 2×10^−5^; Table 1). Corroboratively, the proportions of genes that regulate in the opposite direction to the proposed general model (i.e. the opposite being positively regulating candidate genes in deletions and negatively regulating candidate genes in duplications) are reduced when considering partially duplicated genes as deleted genes.
(XLSX)
