# Supplementary figures and images for: Network Topologies and Convergent Aetiologies Arising from Deletions and Duplications Observed in Individuals with Autism
Source: PLoS Genet. 2013 Jun 6;9(6):e1003523. doi: 10.1371/journal.pgen.1003523 (PMC3675007; doi:10.1371/journal.pgen.1003523)

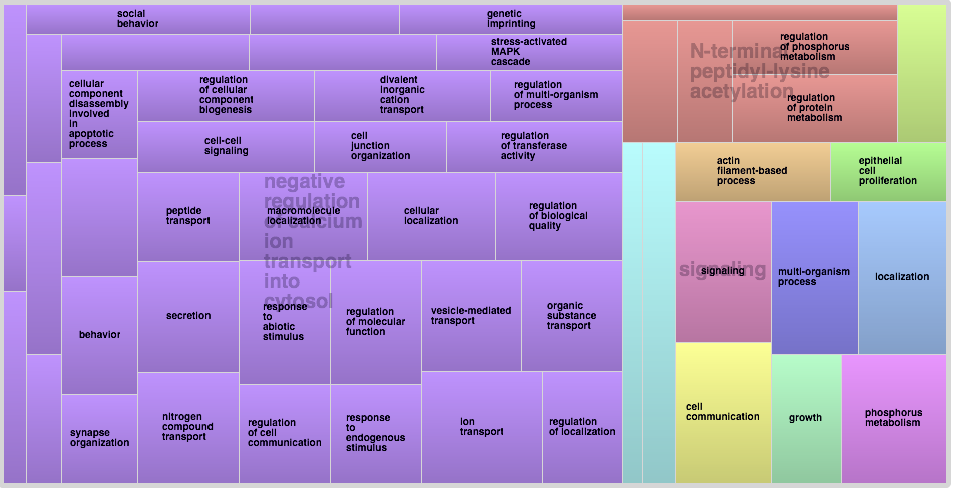

Supplement: Figure S1 — REVIGO-summarised Biological Process Gene Ontology terms enriched within the combined set of ASD dn CNV genes that are associated either with an abnormal synaptic transmission phenotype in the mouse or whose protein product directly interacts with an ASD dn CNV gene that is associated with this phenotype (Supek et al., 2011; The Gene Ontology Consortium et al., 2000). The summarised GO terms are given in full and separately for deletions and duplications in Table S6. (TIFF) [file pgen.1003523.s001.tiff]

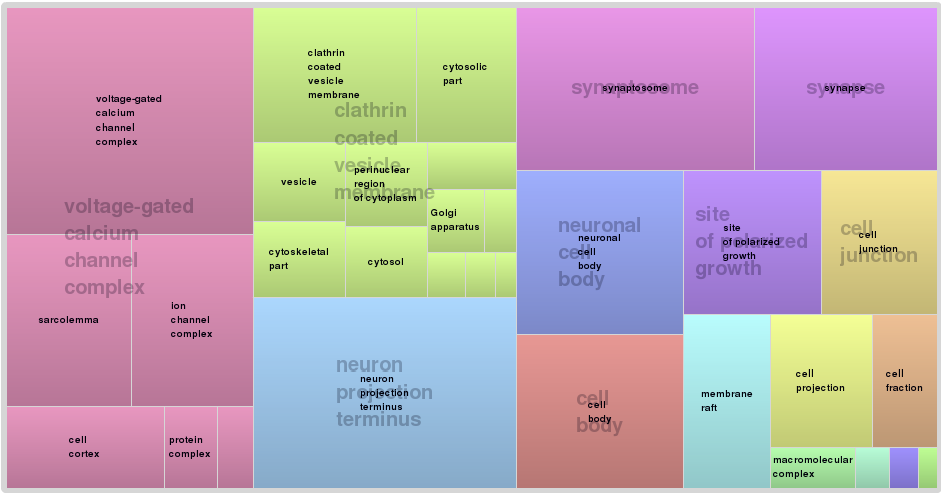

Supplement: Figure S2 — REVIGO-summarised Cellular Location Gene Ontology terms enriched within the combined set of ASD dn CNV genes that are associated either with an abnormal synaptic transmission phenotype in the mouse or whose protein product directly interacts with an ASD dn CNV gene that is associated with this phenotype (Supek et al., 2011; The Gene Ontology Consortium et al., 2000). The summarised GO terms are given in full and separately for deletions and duplications in Table S6. (TIFF) [file pgen.1003523.s002.tiff]

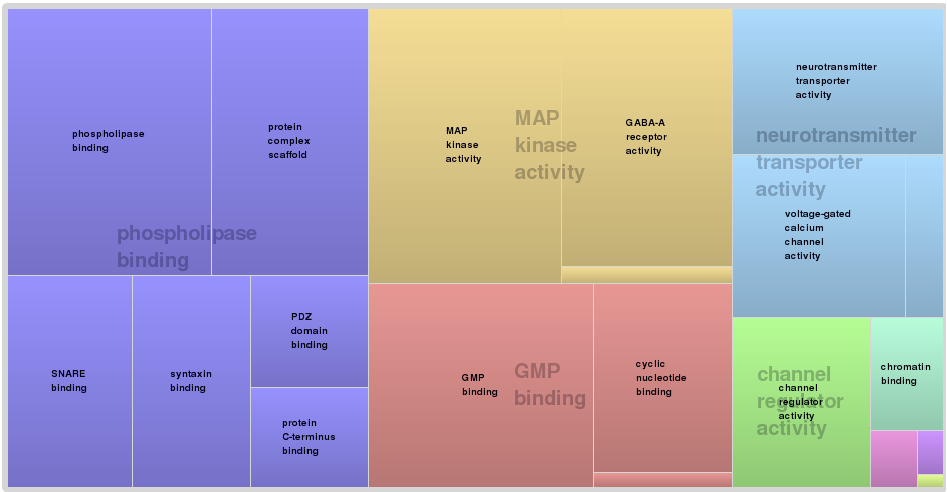

Supplement: Figure S3 — REVIGO-summarised Molecular Function Gene Ontology terms enriched within the combined set of ASD dn CNV genes that are associated either with an abnormal synaptic transmission phenotype in the mouse or whose protein product directly interacts with an ASD dn CNV gene that is associated with this phenotype (Supek et al., 2011; The Gene Ontology Consortium et al., 2000). The summarised GO terms are given in full and separately for deletions and duplications in Table S6. (TIFF) [file pgen.1003523.s003.tiff]

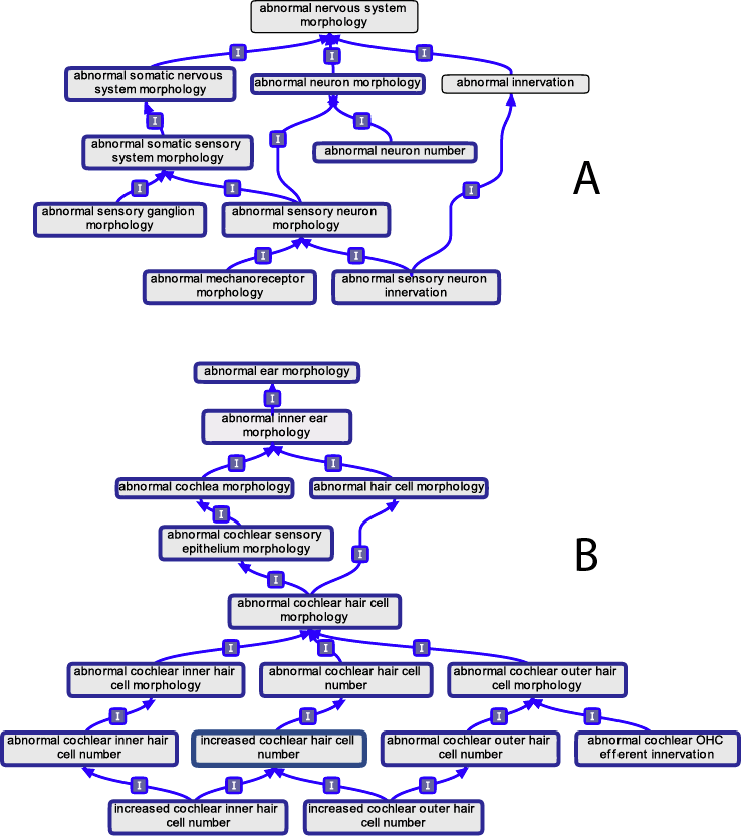

Supplement: Figure S4 — Enriched cochlear and mechanoreception-associated mouse model phenotypes among orthologues of genes overlapped by Gain de novo CNVs from the AGP set. Panel A shows the relevant terms that were identified among Nervous System category phenotypes while Panel B shows the relevant terms that were identified among Hearing/Vestibular/Ear category phenotypes. Relationships between phenotypic terms within the Mammalian Phenotype Ontology are indicated by a blue arrow running from the child term to the parent term. Terms are significantly enriched (BH-adjusted p<5%) if they are shown with a blue border. (TIFF) [file pgen.1003523.s004.tiff]

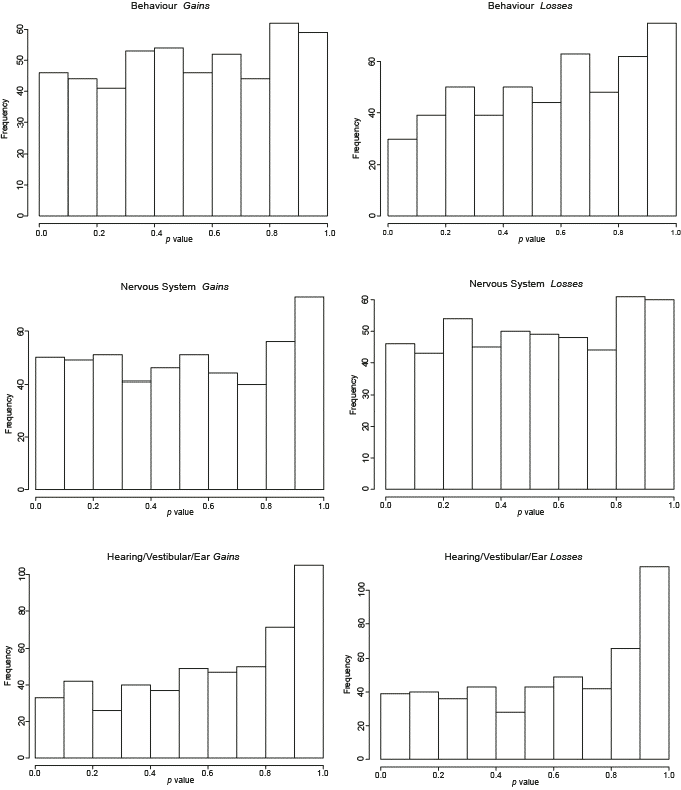

Supplement: Figure S5 — Distributions of p-values for the surfeits of genes associated with 3 phenotypic categories obtained for 500 randomised sets of case-matched CNVRs. 500 sets of genomic regions matched in size and number to each of the Loss and the Gain CNVRs were obtained (see Materials and Methods). For each set, the likelihoods for the observed surfeit of genes associated with a particular phenotypic category, namely Behavior[/Neurological], Nervous System and Hearing/Vestibular/Ear, were recorded and a histogram charted. See also Methods S1. (TIFF) [file pgen.1003523.s005.tiff]

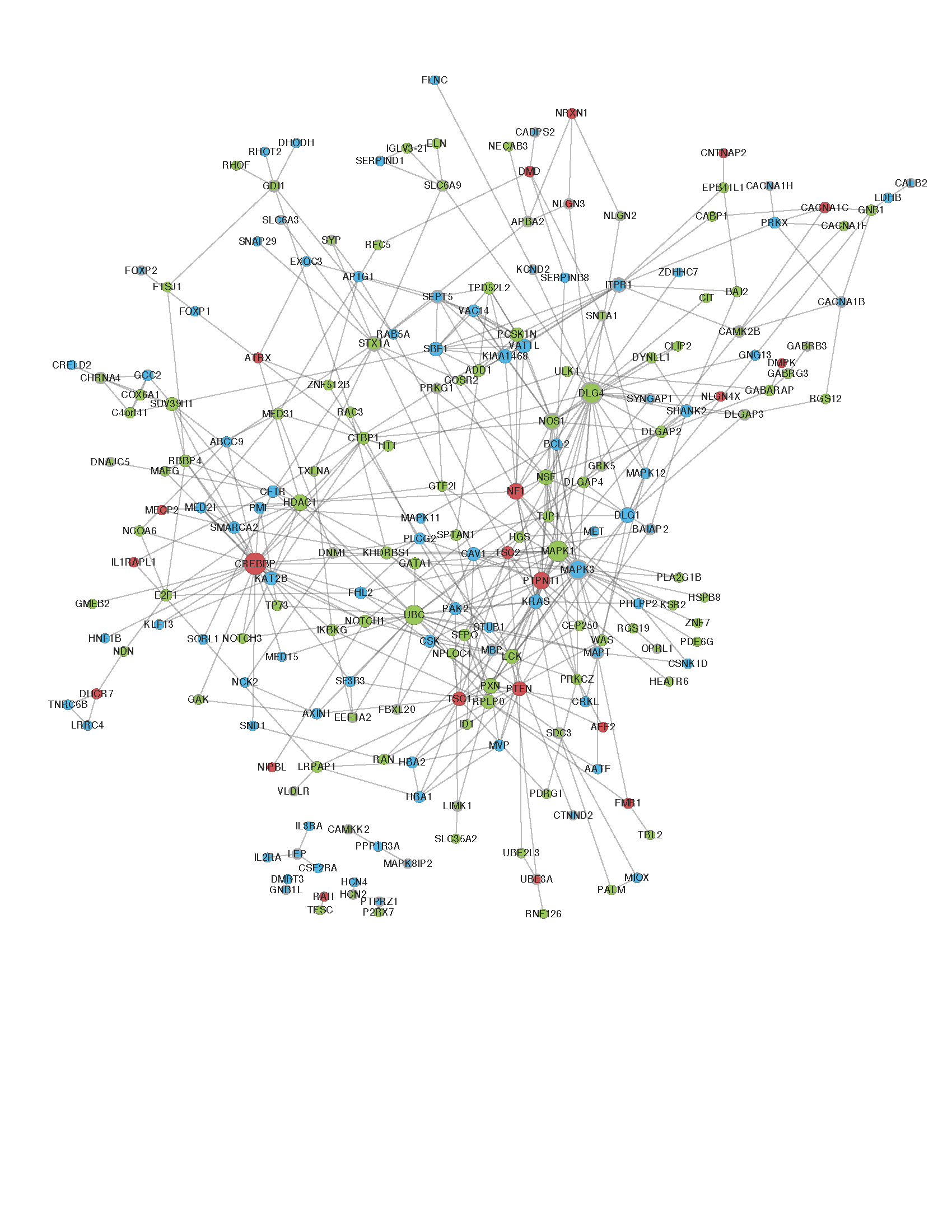

Supplement: Figure S6 — An alternative view of the ASD-associated interaction network shown also in Figure 2 . The network is formed from direct protein-protein interactions between the products of ASD dn CNV genes that are associated with synaptic phenotypes (shown in with thicker grey border), genes previously implicated in ASD (ASD-implicated genes), and other ASD dn CNV genes whose products directly interact with these gene's products. Physical interactions between two proteins are shown as an edge connecting two circles representing each gene. Genes found to be duplicated in autistic patients in this study are shown in green, deleted genes in blue, and ASD-implicated genes in red. (TIFF) [file pgen.1003523.s006.tiff]
