# Supplementary material for: Network Topologies and Convergent Aetiologies Arising from Deletions and Duplications Observed in Individuals with Autism
Source: PLoS Genet. 2013 Jun 6;9(6):e1003523. doi: 10.1371/journal.pgen.1003523 (PMC3675007; doi:10.1371/journal.pgen.1003523)
Supplement: Methods S1 — Statistical analysis and gene assignment. Supporting materials for our method of gene assignment and confirming the null of statistical analysis. See also Figure S5. (DOC) [file pgen.1003523.s007.doc]

**METHODS S1**

***Statistical Analysis and Gene Assignment – Confirming the Null***

Given that many of the CNVs analysed within this study are as small as 30 Kb, it is possible that a CNV that overlaps genic sequence falls wholly within an intron, whilst leaving the spliced transcript intact. Furthermore, even if a given CNV overlapped exonic sequence, that sequence may be alternatively spliced and may only be expressed in a subset of transcripts for that gene. Tissue-specific, and neuronal genes in particular, often have many, large introns and produce many alternative splice forms . To be confident that the expressed coding sequence of a gene is affected, we conservatively required at least one coding exon of every known transcript to be overlapped by a CNV for that gene to be deemed overlapped (**Table S1**). Furthermore, as brain-specific genes are significantly longer than non-brain-specific genes, a higher proportion of brain-specific genes among CNV-overlapped genes may be expected simply by chance (medians 37.1Kb *vs* 25.8Kb respectively, Mann-Whitney U-test *p* = 8 x 10^-4^; brain-specificity defined as in .) However, this expectation does not hold under our CNV gene-assignment approach as the lengths of protein-coding sequence are not significantly different between brain-specific and non-brain-specific genes (medians 455 *vs* 433 residues, respectively, Mann-Whitney U-test *p* = 0.56).

Although our method of gene assignment should be robust against length biases among genes specifically-expressed in the brain (see above), we sought to confirm that our detection of significant enrichments yielded a uniform expectation under the null. To this end, we merged overlapping *Loss* AGP *dn* CNVs and, separately, *Gain* AGP *dn* CNVs to form two sets of copy number variable regions (CNVRs). For each of these sets, we obtained 500 sets of randomly-distributed genomic regions matched in size and number to the CNVRs. Each randomised set was then assigned genes as described above and *p*-values were obtained for the enrichments of genes whose orthologue’s disruption yields a phenotype within particular MGI categories, namely *Behavior/Neurological*, *Nervous System* and *Hearing/Vestibular/Ear* categories. While the resulting distributions of randomised *p*-values are clearly noisy (**Figure S5**), we observe no evidence for a significant surfeit of small *p*-values that might indicate that any significant enrichments we observe in the *dn* CNVs might be obtained simply by chance. Both the apparent noise and the surfeit of random sets that do not overlap any such genes, evidenced by the increased frequency of *p*-values approaching 1, illustrate the scarcity of human genes whose mouse orthologues’ disruption has been phenotyped, particularly for the *Hearing/Vestibular/Ear* phenotypes, and by contrast the gene-richness of the AGP *dn* CNVs (**Figure S5**). Finally, the vast majority of our findings remain if we consider only those genes that are completely overlapped by ASD *dn* CNVs, a requirement that *a priori* penalises longer genes and yields a biologically-significant result.

**Supplemental References**

1. Grosso AR, Gomes AQ, Barbosa-Morais NL, Caldeira S, Thorne NP, et al. (2008) Tissue-specific splicing factor gene expression signatures. Nucleic Acids Res 36: 4823-4832.

2. Webber C, Hehir-Kwa JY, Nguyen DQ, de Vries BB, Veltman JA, et al. (2009) Forging links between human mental retardation-associated CNVs and mouse gene knockout models. PLoS Genet 5: e1000531.
